# Effect of clay nanoparticles on model lung surfactant: a potential marker of hazard from nanoaerosol inhalation

**DOI:** 10.1007/s11356-015-5610-4

**Published:** 2015-11-03

**Authors:** Dorota Kondej, Tomasz R. Sosnowski

**Affiliations:** Central Institute for Labour Protection—National Research Institute, Czerniakowska 16, 00-701 Warsaw, Poland; Faculty of Chemical and Process Engineering, Warsaw University of Technology, Warynskiego 1, 00-645 Warsaw, Poland

**Keywords:** Dust, Nanotechnology, Inhalation, Pulmonary effects, Surfactant

## Abstract

This work investigates influence of different aluminosillicate nanoparticles (NPs) which are found in air in selected workplaces on the properties of the phospholipid (DPPC) monolayer at air–saline interface considered as ex vivo model of the lung surfactant (LS). The measurements were done under physiological-like conditions (deformable liquid interface at 37 °C) for NP concentrations matching the calculated lung doses after exposure in the working environment. Measured surface pressure–area (*π*–*A*) isotherms and compressibility curves demonstrated NP-induced changes in the structure and mechanical properties of the lipid monolayer. It was shown that hydrophilic nanomaterials (halloysite and bentonite) induced concentration-dependent impairment of DPPC’s ability of attaining high surface pressures on interfacial compression, suggesting a possibility of reduction of physiological function of natural LS. Hydrophobic montmorillonites affected DPPC monolayer in the opposite way; however, they significantly changed the mechanical properties of the air–liquid interface during compression. The results support the hypothesis of possible reduction or even degradation of the natural function of the lung surfactant induced by particle–phospholipid interactions after inhalation of nanoclays. Presented data do not only supplement the earlier results obtained with another LS model (animal-derived surfactant in oscillating bubble experiments) but also offer an explanation of physicochemical mechanisms responsible for detrimental effects which arise after deposition of inhaled nanomaterials on the surface of the respiratory system.

## Introduction

Big concern regarding harmful influence of nanoparticles (NPs) on biological membranes (Donaldson et al. 2004; Verma and Stellacci 2006; Shang et al. 2014) triggered numerous investigations focused on physicochemical interactions between nanosized particles and lipid layers (e.g., Wang et al. 2008; Van Lehn et al. 2013; Hoffmann et al. 2014). Phospholipid monolayers are often used as a fundamental ex vivo model of a cell membrane (Brezesinski and Möhwald 2003; Deleu et al. 2014), but they are also considered as a convenient and reliable experimental system for studying interfacial properties of the lung (or pulmonary) surfactant (LS) (Notter et al. 1982; Gradoń et al. 1996; Zasadzinski et al. 2001; Harishchandra et al. 2010; Tatur and Badia 2012; Guzman et al. 2012a). From a toxicological viewpoint, such studies can identify mechanisms of incorporation of nanoparticles with various properties (e.g., size, surface area, surface charge, morphology) into the lipid layer and their impact on structural and mechanical properties of the membrane. The most common method employs the Langmuir film balance to characterize molecular arrangement and rheological properties of interfacial layer based on determined surface pressure–molecular area (π–*A*) relationships. Langmuir balance studies can be assisted by optical methods for in situ visualization of monolayer structure (e.g., Brewster angle microscopy (BAM) and fluorescence microscopy) but also for more detailed analysis after transfer of the film to solid supports (e.g., by atomic force microscope (AFM), Rivière et al. 1994; Vollhardt 1996; Klopfer and Vanderlick 1996; Vie et al. 2000; Guzman et al. 2012a).

Identification of physicochemical interactions between micro/nanoparticles and LS can be the essential step toward preliminary evaluation of the influence of inhaled materials on organisms. Inhaled particles, after their penetration via respiratory airways (trachea, bronchi, and bronchioles) down to alveoli (Heyder and Svartengren 2002; Moskal et al. 2010), settle down on natural liquid layer which contains lipids and specific proteins—the lung surfactant (Zuo et al. 2008; Rugonyi et al. 2008). Properties of particles deposited on the lung surface may be changed by molecules adsorbed from the liquid, and it can result in increased or reduced particle bioavailability (Schleh et al. 2013). On the other hand, if a significant mass of particulates is deposited in the lung surfactant system, a significant amount of material initially present in the liquid can be attached to the particles, so the original composition of the pulmonary liquid can be changed (Sosnowski et al. 2003; Sosnowski 2015). It can result in undesirable health effects not only in respect of breathing mechanics but also of the reduction of pulmonary clearance rate (Gradoń and Podgórski 1989; Sosnowski et al. 2000).

Discussed phenomena still need more investigations because many novel nanomaterials find their industrial application, while their health-related properties remains poorly recognized. For instance, aluminosillicate nanoparticles (nanoclays) became widely applied additives to polymers during manufacturing of new composites with improved mechanical and thermal properties (Mishra et al. 2009; Ray and Okamoto 2003). It is still not clear what the mechanisms of toxicity and the actual risks associated with inhalation of such nanomaterials in the workplace are (Bellmann et al. 1997; Warheit et al. 2010).

In this paper, we investigate the influence of different types of nanoclays on the interfacial properties of a dipalmitoyl phosphatidylcholine (DPPC) monolayer at air–saline interface which can be considered as a basic model of the lung surfactant system and biological membranes. These studies extend our earlier research done for the same NPs interacting with a different LS model in the oscillating bubble experimental system (Kondej and Sosnowski 2013).

## Materials and methods

**Fig. 1:**
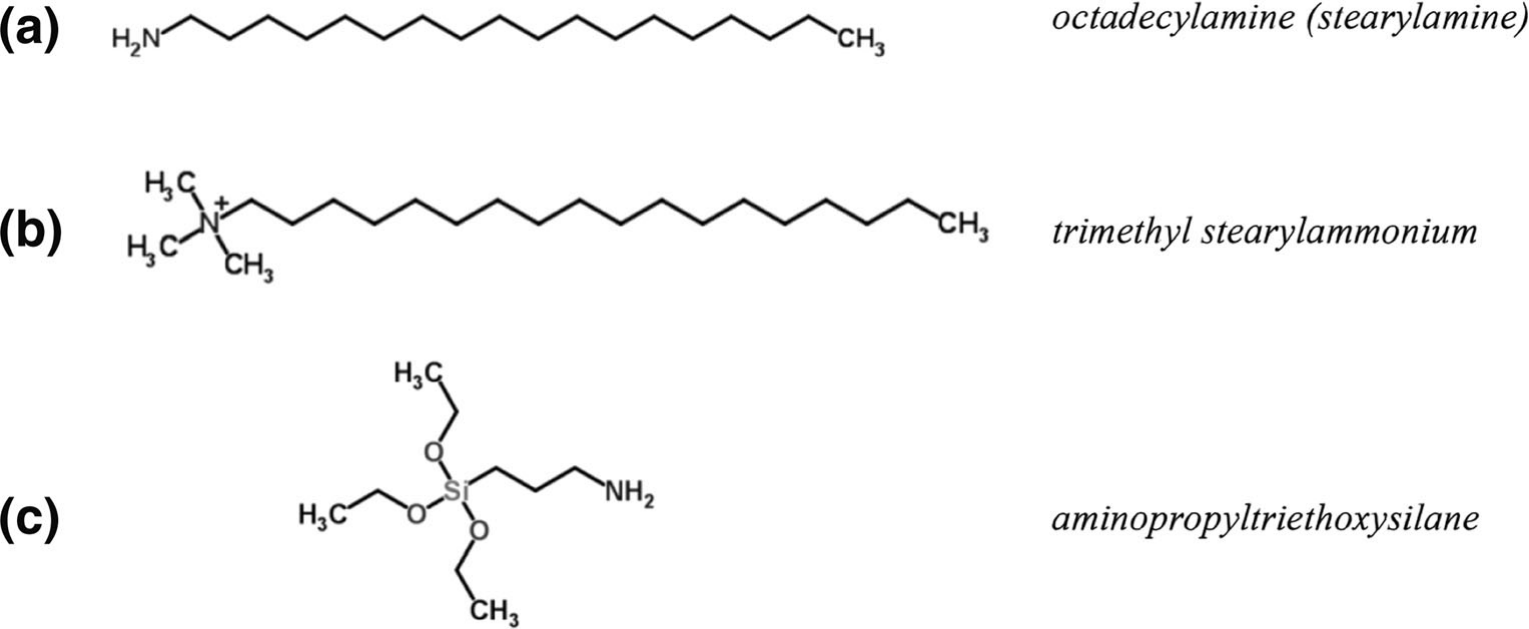
Molecular structure of surface modifiers in M1–M3 hydrophobic nanoclays

1,2-dipalmitoyl-sn-glycero-3-phosphocholine (DPPC, MW = 734.1; 99.5 % pure) and analytical grade chloroform were purchased from Sigma-Aldrich and used as obtained. Sterile saline (0.9 %) was supplied by Polpharma (Poland). Five types of nanopowder aluminosillicates (Table 1) were purchased from Sigma-Aldrich. Halloysite and bentonite (H and B) particles are hydrophilic, while montmorillonites (M1, M2, and M3) are hydrophobic due to their chemical modifications done by the manufacturer in order to obtain a better compatibility with melted polymers, which is important for effective preparation of functional composite materials (Ray and Okamoto 2003; Mishra et al. 2009). The chemical structure of the additives used to obtain hydrophobicity of studied montmorillonites M1, M2, and M3 are shown in Fig. 1.

**Table 1:** Aluminosilicate nanoparticles used in the study^a^

Type	Symbol and name, morphology, particle size, and the specific surface area (SSA)	Chemical composition	CAS No.
H	HN—halloysite	Al_2_Si_2_O_5_(OH)_4_·2 H_2_O	1332-58-7
	Morphology: needles (nanotubes)		
	Particle size: *d* < 100 nm, *L* < 10 μm		
	SSA 25.5 m^2^/g		
B	PGV—bentonite	H_2_Al_2_O_6_Si	1302-78-9
	Morphology: nanoplates		
	Particle size: *h* < 200 nm		
	SSA 67.3 m^2^/g		
M1	I.28E montmorillonite (surface-modified)	montmorillonite 70–75 %	1318-93-0
	Morphology: flakes	trimethyl stearyl ammonium 25–30 %	112-03-8
	Particle size: *h* < 200 nm		
	SSA 9.6 m^2^/g		
M2	I.30E montmorillonite (surface-modified)	montmorillonite 70–75 %	1318-93-0
	Morphology: flakes	octadecylamine 25–30 %	124-30-1
	Particle size: *h* < 200 nm		
	SSA 14.0 m^2^/g		
M3	I.31PS montmorillonite (surface-modified)	montmorillonite 65–85 %	1318-93-0
	Morphology: flakes	octadecylamine15–35 %	124-30-1
	Particle size: *h* < 200 nm	aminopropyltriethoxysilane 0.5–5 %	919-30-2
	SSA 13.5 m^2^/g		

^a^Data regarding particle morphology, size, and SSA based on data by Kondej and Sosnowski (2013)

**Fig. 2:**
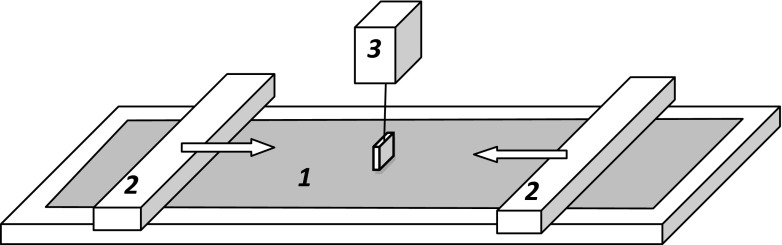
The schematic of Langmuir–Wilhelmy balance: *1*, air/liquid surface covered by DPPC monolayer; *2*, moveable barriers, *3*, pressure sensor with the Wilhelmy plate

Studies were done at 37 °C in thermostated Langmuir–Wilhelmy film balance (KSV, Finland) with polytetrafluoroethylene (PTFE)-coated minitrough (150 × 75 mm) in which symmetrical surface compression was applied (rate of 75 cm^2^/s) by means of two PTFE barriers (Fig. 2). Applicability of this experimental system for studies focused on LS properties comes directly from the fact that these measurements can be done during variations of the air–liquid interfacial area (this resembles physiological situation of alveoli during breathing). In physiological system, the dynamics of respiration requires preservation of surfactant adsorption at liquid interface during compression of the lung surface (i.e., expiration)—it is the essential condition of easy lung inflation during inspiration phase of breathing cycle (Goerke 1992; Gaver et al. 2005).

All parts of the equipment expected to contact with liquids and particles had been carefully cleaned before experiments with ethanol and reverse-osmosis purified water (Puricom, USA). The Wilhelmy plate made of sanded platinum alloy was cautiously cleaned in butane-torch flame. The plate was the measuring element for the surface pressure, which is defined as:

1 $$ \pi ={\sigma}_s-\sigma $$

where *σ*_*s*_ denotes the surface tension of pure liquid (in our case, 0.9 % saline), and *σ* is the actual surface tension of the liquid in the presence of DPPC and nanomaterials. System calibration (i.e., adjustment of zero surface pressure for clean system) was done after gentle aspiration of the air/liquid interface, i.e., the removal of possible surface contaminants.

Nanopowders of each type (as listed in Table 1) were suspended in saline by 5-min ultrasonication required to obtain homogenous dispersions (Kondej and Sosnowski 2013). Studied NPs are not expected to aggregate spontaneously in 0.9 % saline as they typically have a negative zeta potential in solutions with a low to moderate ionic strengths (Baik and Lee 2010). At the beginning of every experiment, the Langmuir trough was filled with NP suspension of defined concentration, and the zero value of π was adjusted immediately. After 15 min required for equilibration, the phospholipid monolayer was formed by gentle spreading of DPPC in chloroform solution (1 mg/ml, applied volume 15 μl) which was applied dropwise on the interface by microsyringe (Hamilton, USA). After complete evaporation of the solvent (approximately 20 min), compression isotherms π–*A* were recorded. The applied amount of the phospholipid was low enough to start experiments from very diluted monolayer (gaseous state). Surface compression measurements were done for all five types of tested nanopowders (H, B, M1, M2, and M3) at three NP concentrations in the liquid phase: 0.25, 0.5, and 1.0 mg/ml. These concentrations correspond to the predicted pulmonary doses of deposited powders during occupational exposure (Kondej and Sosnowski 2013). In order to check if investigated nanopowders exhibit any type of surface activity (as speculated in the previous studies), compression characteristics were also measured in the system without the phospholipid, i.e., when only nanoparticles were present in the liquid subphase. Each experiment was triplicated. The consecutive results were highly reproducible which allowed skipping of the data averaging. Accordingly, all figures with the results show only single lines obtained at the given set of experimental conditions.

Based on measured compression isotherms, surface compressibility at each instant of surface contraction was calculated as:

2 $$ \kappa ={\left(-\frac{d \ln A}{d\pi}\right)}_{T=310K} $$

Compressibility is a rheological property of the interface, and it is equal to the reciprocal of quasi-equilibrium dilational elasticity, *ε* (Guzman et al. 2012b). Both parameters, *κ* and *ε*, characterize the response of the surface layer to mechanical disturbances, i.e., dilation/contraction. Comparison of *κ–π* relationships obtained in systems with different nanoparticles at various concentrations helped to identify influence of nanomaterials on the structure of the phospholipid monolayer and molecular interactions in the superficial film. High values of surface compressibility (i.e., low surface elasticity) correspond to diluted monolayers and low intermolecular interactions, while reduction of *κ* indicates stronger interactions and possible molecular aggregation (condensation) of the surface film. Abrupt rise of compressibility during interfacial compression may also reflect extensive removal of surface-active molecules from the surface layer (similar to the situation observed during monolayer collapse which was, however, not studied here). Reduction of the interfacial area under such conditions leads to very small variations of the surface pressure, indicating downgraded surface-active properties of the monolayer.

## Results and discussion

**Fig. 3:**
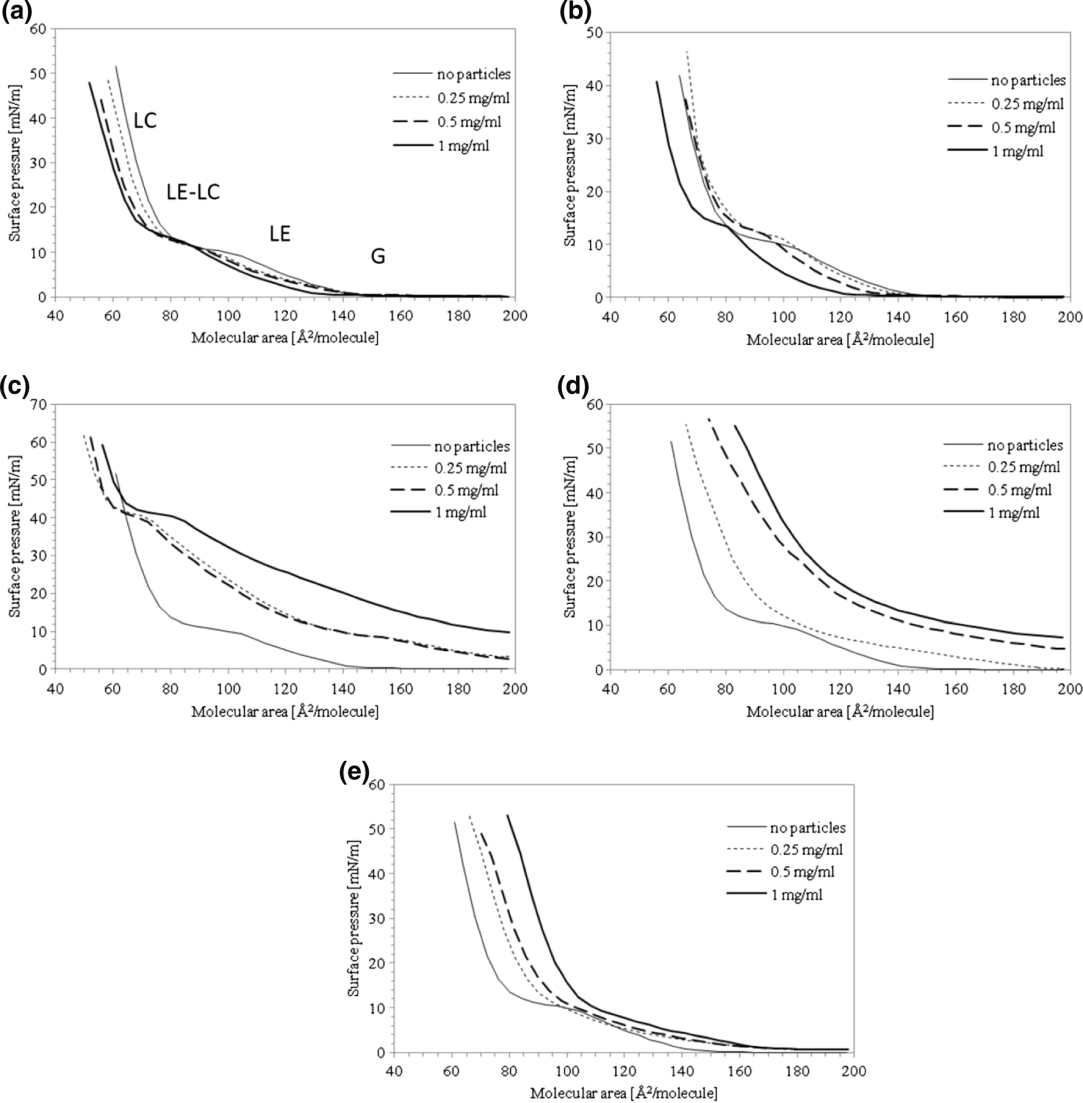
Compression isotherms (37 °C) of DPPC on the surface of 0.9 % saline containing different concentrations of nanoparticles: **a** halloysite H, **b** bentonite B, **c** montmorillonite M1, **d** montmorillonite M2, **e** montmorillonite M3. Molecular area is calculated according to DPPC content only

Compression isotherms of DPPC monolayer on the surface of saline with different concentrations of each type of nanoparticles are depicted in Fig. 3a–e. It should be noted that the molecular area (i.e., the area which may be attributed to a single molecule adsorbed on the interface) is calculated here, taking into account solely the phospholipid content as no information on the real concentration of NPs in the surface film is available.

The molecular organization of DPPC monolayer during surface compression (37 °C, 0.9 % saline subphase) is illustrated in Fig. 3a as *π*–*A* relationship (isotherm), where G corresponds to the gaseous phase, LE to the liquid-expanded phase, and LC to the liquid-condensed phase. LE-LC denotes the coexistence region in which the expanded phospholipid monolayer LE contains dispersed domains of the condensed phase LC (e.g., Vollhardt 1996). Shape of the recorded isotherm is typical for this phospholipid (Guzman et al. 2011; Sosnowski et al. 2012). The coexistence region LE-LC exists at molecular areas of 80–105 Å^2^/molecule which correspond to DPPC surface of 1.58–2.08×10^−6^ mol/m^2^. The surface pressure at this state of monolayer is confined between 10 and 15 mN/m suggesting a moderate degree of intermolecular interactions in the lipid film.

**Fig. 4:**
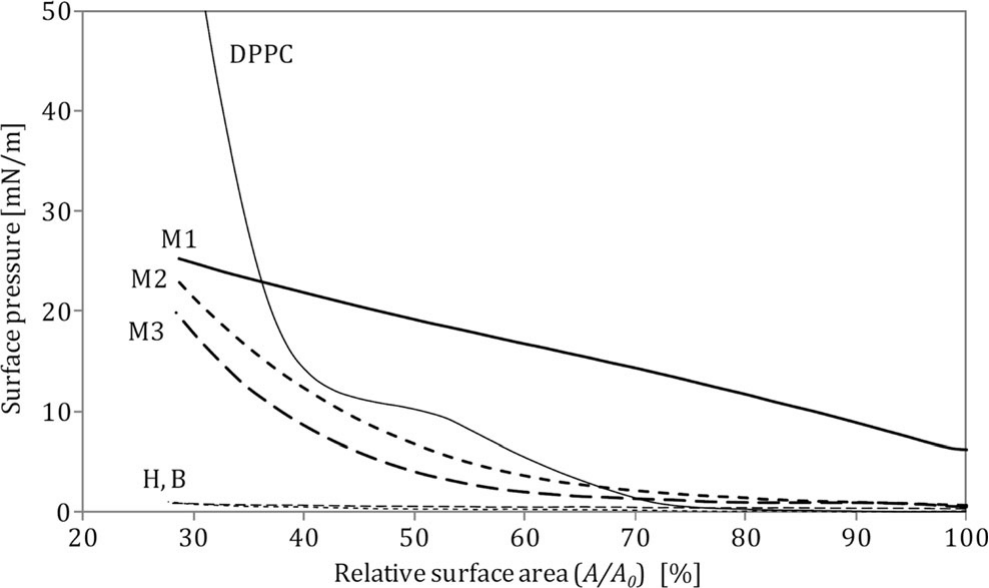
Comparison of surface pressure–area relationships for 1 mg/ml nanoparticles suspensions in 0.9 % saline: **a** halloysite H, **b** bentonite B, **c** montmorillonite M1, **d** montmorillonite M2, **e** montmorillonite M3. Isotherm of DPPC on 0.9 % saline is also shown for a reference

Compression curves obtained in the system which contained only nanoparticles in saline (i.e., system without the phospholipid) are shown in Fig. 4. As already mentioned, these measurements were done to identify surface-active properties either of NPs themselves or of any chemicals which might be liberated from tested nanomaterials after contact with the liquid. In such experiments, the evaluation of molecular area was not possible as neither fixed amount of material could be attributed to the interface nor molecular mass of adsorbed material might be identified. Therefore, these data are presented as the graph of surface pressure versus actual fraction (percentage) of the gas–liquid interfacial area.

**Fig. 5:**
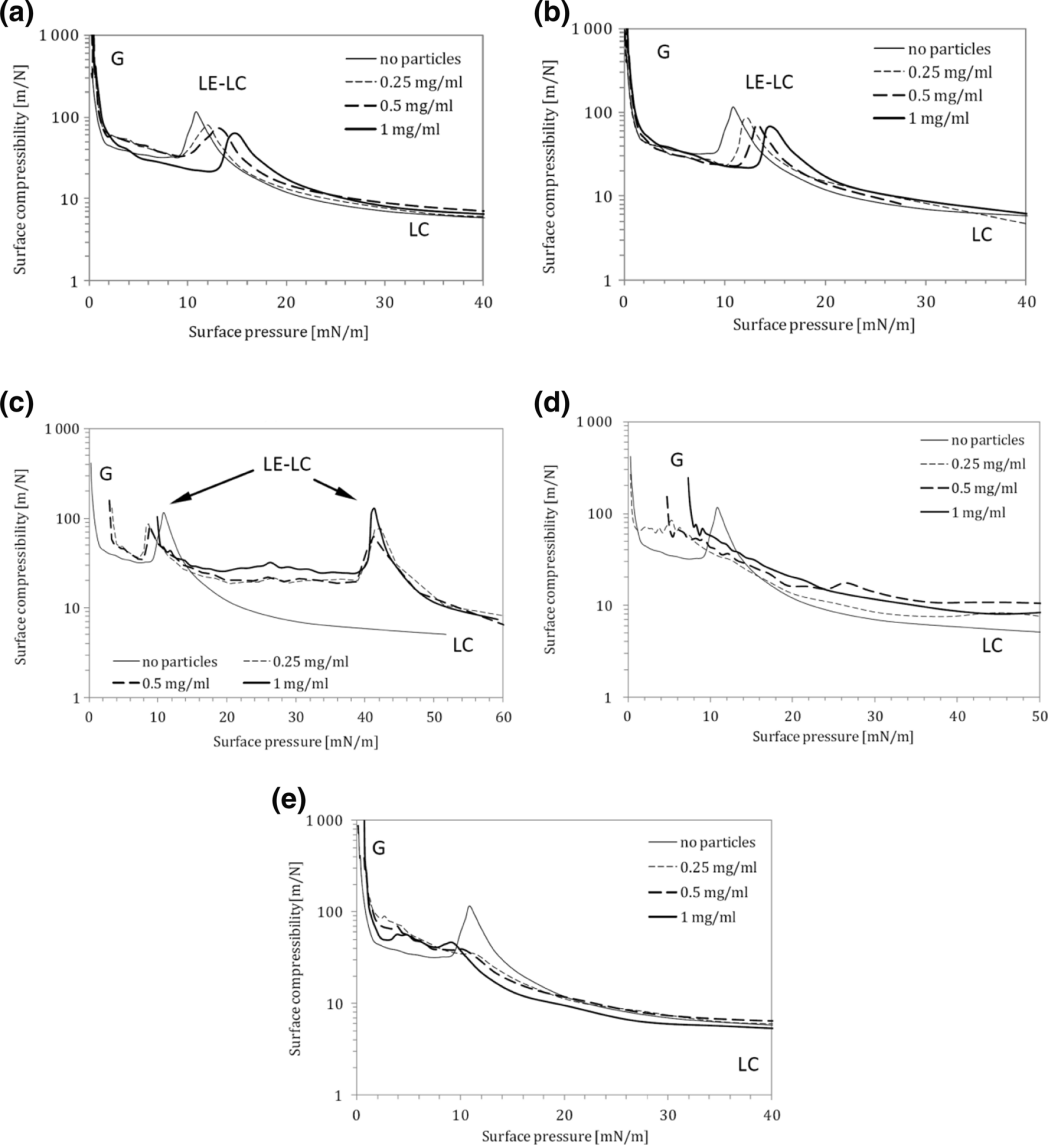
Surface compressibility (37 °C) of DPPC monolayer on 0.9 % saline containing different concentrations of nanoparticles: **a** halloysite H, **b** bentonite B, **c** montmorillonite M1, **d** montmorillonite M2, **e** montmorillonite M3

**Fig. 6:**
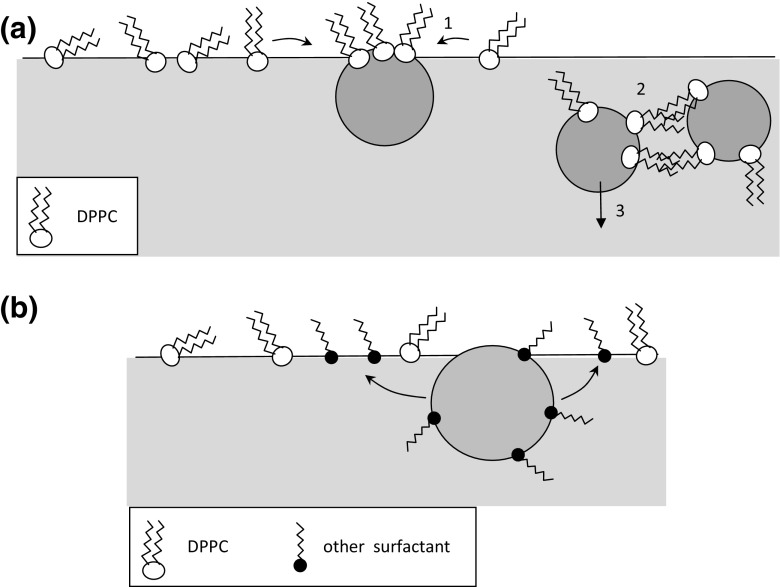
Proposed mechanisms of **a** DPPC exclusion from the monolayer due to binding to hydrophilic NPs: 1—phopsholipid adsorption on NP, 2—clustering of NPs, 3—sinking of the aggregate; **b** competitive adsorption of other surfactants (modifiers) released from functionalized montmorillonite nanoparticles

Figure 5 shows surface compressibility relationships calculated from Eq. 2 based on compression isotherms. The letters printed in Fig. 5 identify the actual state of the monolayer. Addition of halloysite particles (H) to the liquid shifts the *π–A* isotherm to the lower molecular areas (Fig. 3a), suggesting that these NPs can induce a reduction of distance between DPPC molecules, so the monolayer can be packed more densely. Sometimes, such effect can be attributed to a decrease of the lateral size of polar parts of the amphiphile by ionic interactions (Guzman et al. 2011), but in our case—since the ionic strength of the subphase remains constant—a more plausible reason is a decrease of DPPC amount in the surface layer due to preferential adsorption of the phospholipid on nanoparticles. Since halloysite NPs are hydrophilic and not surface active (as demonstrated in Fig. 4), they can bind the polar moieties of DPPC molecules leaving hydrocarbon chains of the amphiphiles exposed outside (Fig. 6a). Accordingly, nanoparticles which become (partly) hydrophobic can aggregate in the liquid phase forming larger clusters which eventually sink. Therefore, some amounts of the phospholipid leave the gas–liquid interface, and it is reflected by horizontal shift of the *π–A* isotherm. The extent of observed dislocation of *π–A* curves in Fig. 3a is proportional to NP concentration which is consistent with the proposed mechanism. This phenomenon can be also detected by analyzing surface compressibility curves (Fig. 5a), where *κ*_max_, representing the middle of the coexistence region LE-LC, is successively shifted toward higher surface pressures with increasing content of NPs in the liquid. This confirms that the coexistence region is attained at higher degrees of film compression.

Dependence of the π–*A* isotherm on addition of bentonite (B) nanoparticles (Fig. 3b) is slightly different from the one found for halloysite, although properties of both NPs are similar (both are hydrophilic and not surface active; see Fig. 4). In fact, almost no change in compression characteristics is observed at low bentonite concentrations, while a noticeable shift of the isotherm is found for high particle content (1.0 mg/ml). It is an unexpected result since bentonite NPs have higher specific surface area than halloysite NPs (67.3 vs. 25.5 m^2^/g; see Table 1), so according to the postulated mechanism, higher phospholipid adsorption on B particles was expected. It should be noted, however, that the specific surface area determined by nitrogen adsorption (Kondej and Sosnowski 2013) reveals the total area of all pores in the structure of dry material, while not all these pores may be available for adsorption of DPPC molecules due to steric restrictions. Additionally, H and B nanoparticles differ in shape (plate-like bentonite particles, nanotubes of halloysite; see Table 1), and this factor can affect their surface properties leading to dissimilar behavior, especially if other effects (such as disaggregation or swelling) come into play in the aqueous environment. Interestingly, the divergence demonstrated by *π–A* relationships is not reflected by the shape of compressibility curves (Fig. 5a, b). It can be simply explained by mathematic principles (*κ* is calculated as a derivative of the primary relationship).

Observed influence of two types of hydrophilic nanoclays (i.e., H and B) on DPPC monolayer differs from the effects reported for other types of hydrophilic nanoparticles, such as silica (Guzman et al. 2011) or carboxyl-modified polystyrene (Farnoud and Fiegel 2012). The referred studies suggested that spherical nanoparticles might be incorporated in the interfacial film, and they decrease the available interfacial area after forming partially hydrophobic DPPC–NP complexes (Guzman et al. 2011). In effect, an increase of *π* was observed at higher values of molecular area, i.e., at lower surface concentration of DPPC. Such complexes are also expected to influence intermolecular interactions in the monolayer; therefore, they may alter the process of formation of condensed domains of LC phase during compression (Guzman et al. 2011; Farnoud and Fiegel 2012). Indeed, *κ*–*π* relationships obtained in this work for H and B nanoparticles confirm monolayer modification during compression in the coexistence region, which fully agrees with the discussed concept. The squeezing out of the lipid–NP complexes from the interface proposed as the explanation of our results was also postulated by other authors (Guzman et al. 2012b; Farnoud and Fiegel 2012). By studying surface pressure hysteresis during compression–expansion cycle, Farnoud and Fiegel (2013) also indicated that NPs which were displaced together with the phospholipid molecules from the interface can slowly return to the surface layer. Such removal of NPs from the liquid surface may be amplified in our studies by nonspherical shape of nanoclay particles and possible particle clumping after phospholipid adsorption on their surface. Support for the hypothesized DPPC adsorption on the surface of mineral particles is provided by toxicological studies. Biological assays showed suppression of toxicity of clay particles after their incubation with this phospholipid (Gao et al. 2001), which was attributed to binding of the head group of DPPC to the protonated aluminol group (Al–OH^2+^) present on the surface of clays (Snyder and Madura 2008).

Compression of DPPC monolayer in the presence of surface-modified (hydrophobized) montmorillonites M1–M3 produces substantially different results from those obtained for hydrophilic H and B particles. It is seen in Fig. 3c, d that M1 and M2 particles increase surface pressure in the system even without surface compression, which suggests that they demonstrate some surface activity. This is also confirmed by the results presented in Fig. 4 and can be explained by the mechanism where either NPs or chemicals liberated (eluted) from nanomaterials adsorb at the air–liquid interface, leading to an increase of the measured surface pressure (Fig. 6b). In case of M1 nanoparticles at concentrations 0.25 and 0.5 mg/ml, the recorded *π*–*A* curves are vertically shifted by approximately 5 mN/m even without compression, and stronger shift is observed at higher NP concentration (by more than 10 mN/m at 1.0 mg/ml, without surface compression; Fig. 3c). Consequently, a visible shift of phase transitions toward higher surface pressures is observed during surface contraction. It is visible also in *κ*–*π* relationships (Fig. 5c) where *κ*_*max*_ occurs at approximately 40 mN/m (instead of 14 mN/m observed for DPPC only), although, the nominal value of *κ*_*max*_ remains practically unchanged. Results obtained for M1 nanoparticles at the concentration 1.0 mg/ml in the subphase without DPPC (Fig. 4) confirm that the initial increase of the surface pressure is caused solely by NPs and/or eluted surface-active chemicals which are adsorbed at the air–liquid interface. This can be explained by the fact that the surface-modifying agent present in M1 particles, i.e., octadecylamine (Fig. 1a), is capable of forming Langmuir monolayers and indicate surface activity (Stine and Stratmann 1992; Tsai and Lee 2009).

Concentration-dependent shift of the *π*–*A* relationship is also found for M2 particles (Fig. 3d), but this isotherm is additionally characterized by the complete disappearance of coexistence (LE–LC) plateau. It is also clearly illustrated by vanishing *κ*_max_ on the surface compressibility curves (Fig. 5d). This effect can be rationally explained assuming incorporation of these NPs (or eluted surface-active compound) into the DPPC film. During surface compression, the molecular reorganization of the surface layer together with the exclusion of NPs leads to disturbed formation of condensed phospholipid domains. Such changes of monolayer arrangement seem to be specific for the surface modifier present in M2 nanoparticles (trimethyl stearyl ammonium cation). Surface activity of this compound is evident considering its chemical structure (Fig. 1b). It was also confirmed by the experimental results obtained in the M2–saline system without DPPC (Fig. 4d).

In contrast to the results obtained for M1 and M2, there is no vertical shift of *π*–*A* relationships recorded in the system with M3 nanoparticles (Fig. 3e). The LE-LC coexistence region is undetectable, and this observation corresponds to the absence of *κ*_*max*_ on compressibility curves (Fig. 5e). However, the isotherms are horizontally shifted toward higher values of surface area suggesting incorporation of NPs into the interfacial film and formation of hydrophobic complexes with the lipid, similarly as described by Guzman et al. (2011). By contrast to complexes formed by the phospholipid and hydrophilic NPs (halloysite or bentonite), hydrophobic M3 nanoparticles should attract and bind hydrocarbon tails of DPPC molecules. It is also evident that the surfactants contained in this nanomaterial do not interact individually with the phospholipid as seen from low surface activity recorded in the system without DPPC (Fig. 4)—it is the weakest of all studied surface-modified montmorillonites. Since M3 nanoparticles were modified by two surface-active compounds, it seems that the presence of 3-aminopropyltriethoxysilane (Fig. 1c) suppresses surface activity of the second hydrophobic modifier (antagonistic interaction).

All presented results show that selected types of nanoclays, which differ in particle shape, specific surface area, and surface composition/properties, induce variable responses of DPPC monolayer under simulated physiological conditions. The general findings from the presented experiments are in a qualitative agreement with the results obtained earlier by Kondej and Sosnowski (2013, 2014) using oscillating bubble and pendant drop. In the referred studies, higher surface activity was observed for all surface-modified aluminosillicate NPs (M1, M2, M3), while the opposite effect was found for halloysite and bentonite nanoparticles (H and B). It must be noted though that the referred data cannot be directly compared to Langmuir-balance results as the former were obtained for a complete, i.e., multicomponent animal-derived lung surfactant, which contains DPPC in the mixture with other natural surface active compounds (lipids and proteins). In addition, surface deformations applied in the oscillating bubble studies were significantly beyond the linear regime which disables direct evaluation of surface rheological quantities that might be compared to *κ* determined in the current research. Nevertheless, amplification of surface tension–surface area hysteresis found for M1, M2, and M3 nanoparticles in the reported oscillating bubble experiments (Kondej and Sosnowski 2013) indicates the domination of viscous over elastic components in the mechanical response of air–liquid interface to dilatational deformation. This effect can be directly linked to the increased value of *κ* (i.e., reduced surface elasticity) of DPPC monolayer after interactions with the studied nanoparticles in this study. Similar changes of rheological characteristics of air–liquid interface in the real lung surfactant system can affect the mechanics of breathing (Goerke 1992).

Extending our discussion beyond the data obtained for the NPs considered in this study, it should be noticed that the results obtained in experiments of this kind may dependent on additional factors. The important one is, e.g., the method by which particles are introduced into the studied air–liquid system. As shown by Farnoud and Fiegel (2013) in their recent work, nanoparticles suspended in the liquid interact differently with freshly formed phospholipid surface film than NPs injected to the subphase after the monolayer had been already formed. This introduces additional questions in discussion of mechanisms of NP interactions with the monolayer hampers the possibilities of confronting results obtained by different groups. In the real life LS system, all inhaled nanoparticles are carried into the lungs as aerosol, so they land on the liquid surface before they are translocated to the subphase. Unfortunately, this situation is difficult to reconstruct in physicochemical settings due to the common requirements of cleanliness (dust-free environment), so simplified experimental approaches are typically used. A proposition of a new exposure system for studies of that type was proposed very recently (Farnoud and Fiegel 2015).

## Conclusions

This research demonstrates that the fundamental, surface-active properties of DPPC monolayer—considered here as basic model lung surfactant and biological membrane—are modified by nanosized aluminosillicate particles in a concentration-dependent manner, and that the observed effects are highly specific for different types of tested nanomaterials. Observed effects of hydrophilic nanomaterials (bentonite and halloysite) suggest that lipid molecules are adsorbed on the surface of mineral particles, and lipid–NP complexes are formed which can further aggregate. This leads to partial removal of the phospholipid from the interface and indicates destructive effect of hydrophilic NPs on the DPPC film. It can be argued that similar mechanisms disturb phospholipid properties and functions in the lung surfactant system in vivo. Reduced amounts of the phospholipids at the air–liquid interface means impaired reduction of the surface tension during alveolar surface compression associated with exhalation of air during breathing, so it affects breathing mechanics. Surprisingly, in spite of a larger apparent surface area, bentonite particles have lesser effect on DPPC monolayer than halloysite NPs. This can be attributed to differences in shapes of these nanoparticles (nanoplates vs. nanotubes, respectively) and in the surface area actually available for phospholipid adsorption in their porous structure. Two types of hydrophobic (modified) flake-like montmorillonites (M1 and M2) affected DPPC monolayer in the opposite way, inducing apparent increase of the surface pressure in the experimental system. It is postulated that such effect is caused by surface-active properties of NPs and/or of chemical modifiers which might be released from the particles after their contact with the liquid. Even if surface-active properties of the system become enhanced, they can be considered undesired and damaging to the gentle balance of mechanical forces naturally operating in the lungs due to the LS. There is a literature evidence that appearance of foreign surface-active compounds in the respiratory system leads to serious health conditions (Rao and Das 1994; Hannu et al. 2012).

Results of presented research support and partially explain previous findings obtained in the oscillating bubble experiments for the whole (multicomponent) lung surfactant. The physical picture of interactions between nanoparticles and the phospholipid at the air–liquid interface which is postulated in the current work may help to explain physical phenomena and the subsequent biochemical processes occurring in the respiratory system after breathing air contaminated by similar nanoparticles.
